# Comparative study of normal and sensitive skin aerobic bacterial populations

**DOI:** 10.1002/mbo3.138

**Published:** 2013-10-23

**Authors:** Mélanie Hillion, Lily Mijouin, Thomas Jaouen, Magalie Barreau, Pauline Meunier, Luc Lefeuvre, Elian Lati, Sylvie Chevalier, Marc G J Feuilloley

**Affiliations:** 1 Laboratory of Microbiology Signals and Microenvironment EA 4312, University of Rouen27000, Evreux, France; 2 BIO-EC Laboratory92140, Longjumeau, France; 3 Dermatologic Laboratory URIAGEBP50 92404, Courbevoie Cedex, France

**Keywords:** Dysbiosis, host bacteria interactions, mass spectrometry, sensitive skin syndrome, skin bacteria

## Abstract

The purpose of this study was to investigate if the sensitive skin syndrome, a frequent skin disorder characterized by abnormal painful reactions to environmental factors in the absence of visible inflammatory response, could be linked to a modification in the skin bacterial population. A total of 1706 bacterial isolates was collected at the levels of the forehead, cheekbone, inner elbow, and lower area of the scapula on the skin of normal and sensitive skin syndrome-suffering volunteers of both sexes and of different ages. Among these isolates, 21 strains were randomly selected to validate in a first step the Matrix-Assisted Laser Desorption/Ionization (MALDI)-Biotyper process as an efficient identification tool at the group and genus levels, by comparison to API® strips and 16S ribosomal RNA gene sequencing identification techniques. In a second step, identification of the skin microbiota isolates by the MALDI-Biotyper tool allowed to pinpoint some differences in terms of bacterial diversity with regard to the collection area, and the volunteer's age and gender. Finally, comparison of the skin microbiota from normal and sensitive skin syndrome-suffering volunteers pointed out gender-related variations but no detectable correlation between a phylum, a genus or a dominant bacterial species and the sensitive skin phenotype. This study reveals that there is no dysbiosis of aerobic cultivable bacteria associated with the sensitive skin syndrome and further demonstrates that the MALDI-Biotyper is a powerful technique that can be efficiently employed to the study of cultivable human skin bacteria. To our knowledge, this is the first study focusing on bacteria in the sensitive skin syndrome. These results are of potential importance for pharmaceutical and cosmetic industries, which are looking for new strategies to treat this multiparametric disorder.

## Introduction

Skin is a complex ecosystem including yeasts, fungi, bacteria, and viruses (Kong and Segre 2012). One billion bacteria are associated with each square centimeter of skin (Grice et al. 2008) and almost 25% of this population is located deeply into the dermis (Lange-Asschenfeldt et al. 2011). This bacterial population is directly interacting with keratinocytes, and bacteria play a central role in skin physiology and diseases (Rosenthal et al. 2011).

*Staphylococcus aureus* and *Propionibacterium acnes* are major determinants of acne (Coenye et al. 2008). *S. aureus* is not only responsible for folliculitis, furunculosis, cellulitis or impetigo but also many other bacterial species, including *Streptococcus pyrogenes*, *Pseudomonas aeruginosa* or even *Helicobacter pylori* have been associated with skin infections (Laube 2004; Bhagavatula and Powell 2011). However, the origin of many skin diseases is multifactorial and in some cases, such as the sensitive skin syndrome, the role of bacteria remains to be clarified. The sensitive skin syndrome by itself has been discussed for a long time and it is only recently that, with a more precise definition and the development of standard tests, it was clearly defined (Fluhr et al. 2008). Generally, it is characterized by an abnormal sensory reaction, including itch and pain, to environmental aggressions such as ultraviolet (UV), heat, cold, pollutants or cosmetics, but in the absence of visible signs of inflammation (Farage and Maibach 2010). The prevalence of this syndrome is high, reaching very diverse degree of intensity in 50% of the western female population (Misery et al. 2009). Ceramides, which are important skin barrier components (Novotný et al. 2010), are decreased in sensitive skin patients (Cho et al. 2012) and, as observed in atopic dermatitis (Elias and Schmuth 2009), an alteration of the skin barrier function allows an abnormal bacterial colonization. Although antibiotics and germicides are not generally very efficient, other treatments leading to reduce the skin bacterial population by limiting bacteria growth or biofilm formation generally improve the symptoms (Masako et al. 2005; Berardesca et al. 2009). These data suggest that the sensitive skin syndrome should be linked to a local dysbiosis but until now the bacterial population of sensitive skins has not been investigated.

In a first attempt, and in order to focus on metabolically active microorganisms, we used a culture-based technique associated with a Matrix-Assisted Laser Desorption/Ionization (MALDI) mass spectrometric bacterial identification technique (Biotyper system) to compare the cultivable aerobic bacterial populations on normal and sensitive skin patients. This culture-based approach was also justified by the need to collect reference human skin strains for further metabolic studies. The collection procedure was carefully standardized. These bacteria were classified by groups and species in order to point out possible variations associated with the skin sensitive syndrome.

## Material and Methods

### Collection procedure

Bacterial strains were collected following a strict procedure as this step is essential for the validity of the study (Rosenthal et al. 2011). As Grice et al. (2008) showed that there is no difference between the skin microbiome obtained from swab, scrabe or punch biopsy, bacterial isolates were collected here by swabbing under control of the CRO Bio-EC (Longjumeau, France) and in agreement with French and EU Ethic guidelines (ARS Biomedical Research Agreement N 2012-12-010, Bioethic Agreement DC-2008-542).

The collection technique was established in a preliminary study. Three different swabs (cotton wool wad with wood rod, viscose wad with plastic rod or carded cotton wad with wood rod) and two humidification solutions (NaCl 0.15 mmol/L in sterile water supplemented or not with 0.1% Tween20) were tested. Bacteria were collected on the skin of three female donors at 1 week interval. None of the subject was exposed to antibiotic treatment for at least 1 month before the experiment. They were not allowed to clean or to use any cosmetic 24 h before bacterial collection. After collection, each swab was immediately placed in a sterile tube containing 2 mL of the same humidification solution. The rod was cut and the tubes were vortexed for 10 sec. Swab fibers and fragments were removed by low speed centrifugation (1 min; 80*g*) and an aliquot (100 μL) was plated onto Tryptone Soy Agar medium (TSA). Petri dishes were incubated at 28°C and 37°C for 72 h. The number of visible colonies was noted as + (<10 colonies/petri dish), ++ (<50 colonies/petri dish) or +++ (≥50 colonies/petri dish). As shown in Table 1, the highest number of isolates was recovered using cotton wool wad with wood rod swabs humidified with 0.15 mol/L NaCl and 0.1% Tween20 in sterile water. These swabs were used for the rest of the study.

**Table 1:** Abundance of bacterial isolates collected from an identical skin area (inner elbow) using different swabs and humidification solution

ROD	Wood				Plastic	
		
WAD	Carded cotton (hand-made)		Cotton wool (commercial)		Viscose (commercial)	
Growth (°C)	28	37	28	37	28	37
0.15 mol/L NaCl	+	+	++	+++	++	++
0.15 mol/L NaCl + 0.1% Tween 20	+	+	+++	+++	++	++

The number of isolates is noted as + (<10 colonies/petri dish), ++ (<50 colonies/petri dish) or +++ (≥50 colonies/petri dish). Each sampling condition was tested by growing bacterial isolates at 28 and 37°C.

For the study, three groups (each one formed of three male patients and three female patients) were selected. The size of these groups (six patients) was limited by the difficulty to form a homogenous sensitive skin panel but it remains in the same range as in previous studies on the human skin microbiote (5 for Grice et al. 2008; 6 for Gao et al. 2007; 11 for Capone et al. 2011 or Staudinger et al. 2011). The first group was formed of 20- to 35-year-old normal skin patients exempt of any known infectious disease. The second group was formed of 50- to 65-year-old normal skin patients also without known infectious disease. The third group was formed of 20- to 35-year-old patients presenting a sensitive skin phenotype, with an atopic background but not in crisis period and/or under treatment. These patients were also selected on the basis of a widespread sensitive skin syndrome affecting both face and back (including scapula). The sensitive skin phenotype was established on the basis of the adverse sensory response to the lactic acid sting test (LAST) (Robinson and Perkins 2001).

Samples were collected at the levels of the forehead, cheekbone, inner elbow (antecubital fossa), and lower area of the scapula. For swabbing, the surfaces (4 cm^2^ at the level of the forehead, cheekbone or inner elbow and 20 cm² at the level of the scapula) were determined by sterile cardboard masks and these areas were gently rubbed 20 times in horizontal, vertical, and transversal directions. Each patient was sampled on the right and left sides of the body by two different investigators generating a total of 12 collection samples for each group. Swabs were immediately transferred into collection tubes and processed as previously described.

### Bacterial culture

A volume of 100 μL of bacterial suspension was plated onto normal and blood supplemented (5%) Trypticase Soy Agar medium (TSA). Petri dishes were incubated at 28°C and 37°C for 72 h to allow the development of psychrotrophic and mesophilic bacteria.

### Comparison of bacterial identification techniques

The optimal growth temperature (28 or 37°C), the colony macroscopic aspect and the hemolytic activity on blood TSA of each isolate were noted. The respiratory metabolism was tested by deep inoculation in meat–liver glucose agar. The morphology, mobility, and Gram stain of each isolate were also determined. The catalase and oxidase activities were tested using hydrogen peroxide 3% solution and oxidase strips (Merck, Darmstadt, Germany), respectively.

After these orientation tests, a randomized group of 21 isolates was submitted to metabolic identification using API® strips (BioMerieux, Craponne, France): 20E for Enterobacteriaceae, 20NE for non-Enterobacteriaceae, ID32 Staph for Staphylococcaceae and Micrococcaceae, 20Strep for Streptococcaceae, Coryne for Corynebacterium, 50CH for Bacillaceae. The same isolates were also submitted to sequencing of the 16S ribosomal RNA genes using Fwd OL (GTGTAGCGGTGAAATGCG) and Rev OR (ACGGGCGGTGTGTACAA) universal primers, as described by Bodilis et al. (2004), which were aligned with reference sequences using Clustal W version 1.83 with default parameters (http://www. ebi.ac.uk/clustalw/). Finally, these isolates were treated for bacterial identification by MALDI mass spectrometry using an algorithmic method of comparison of the total proteome.

### Mass spectrometric MALDI-Biotyper bacterial identification

Bacteria were identified by analysis of the total proteome using an Autoflex III Matrix-Assisted Laser Desorption/Ionization-Time-Of-Flight mass spectrometer (MALDI-TOF) (Bruker, Marcy-l'Étoile, France) coupled to the MALDI-Biotyper 3.0 algorithmic system for microbial identification. A colony from each isolate was picked, dispersed in pure water, and centrifuged (1 min at 16,000*g*). The pellet was then resuspended in 70% ethanol/water (v/v) and centrifuged again for 1 min at 16,000*g*. The pellet was mixed with 70% formic acid and an equal volume of acetonitrile was added. The suspension was centrifuged (2 min at 16,000*g*) and 1 μL of the supernatant was spotted onto a MALDI target plate and dried at room temperature. The spot was then overlaid with 1 μL of matrix solution (10*g*.L^−1^ *α*-cyano-4-hydroxycinnamic acid in 50% acetonitrile, 2.5% trifluoroacetic acid) and dried again. Mass spectra were automatically generated using the Autoflex III MALDI-TOF/TOF operated in the linear mode over a mass range from 2000 to 20,000 Da. The instrument was calibrated using a Bruker bacterial test standard. For each sample, 600 spectra, obtained under 200 Hz at 30% YAG laser power, were pooled and the resulting spectrum was analysed using the MALDI-Biotyper 3.0 system (Bruker). The software generated score values representing the probability of correct identification of the microorganism. This score was calculated on the basis of the number of signals in the reference spectrum that closely matched those in the unknown spectrum, the number of signals in the unknown spectrum closely matching to partners in the reference spectrum and the symmetry of the matching signal pairs. Below a score value of 1.7, a spectrum was rated nonidentifiable. Between 1.7 and 2.0 the genus identification was considered sure. The species identification was considered valid for score values >2.0.

## Results

### Comparison of the three bacterial identification techniques

For the 21 randomly selected isolates, the score value by mass spectrometry was above 2.0 allowing a comparison between the three identification techniques. The performances of the techniques were almost the same at the genus level (20 isolates classified in the same groups between the MALDI-Biotyper and 16S RNA sequencing techniques and 19 between MALDI-Biotyper and API strip identification) (Table 2). At the species level the percentage of correlation among the three techniques was lower with a mean of 17 isolates identified in the same species between the MALDI and API or 16S identification techniques. Considering the substantial saving of time and low cost (when the equipment is available) of the MALDI-Biotyper technique we decided to apply this approach to the study of bacterial diversity on normal and sensitive skins. Five of these isolates identified under the same species by the three techniques were included in the library of the laboratory under reference numbers MFP01–05.

**Table 2:** Comparison of 21 randomly selected skin bacterial isolates identified by use of API® strips, MALDI-TOF Biotyper and 16S rRNA gene sequencing

Isolate no	API® strips identification	MALDI-Biotyper identification	165 rRNA identification	LMSM Ref.
2.N.F2.PG.28.2	Kocuria sp.	*Kocuria rhizophila*	Kocuria sp.	
2.N.F3.FG.28.1	***Staphylococcus aureus*** **or** ***epidermidis***	***Staphylococcus warneri***	***Staphylococcus aureus*** **or** ***epidermidis***	
2.N.F3.PD.37.4	*Proteus mirabilis*	*Proteus mirabilis*	*Proteus mirabilis*	
2.N.M2.PD.37.4	*Staphylococcus epidermidis*	*Staphylococcus epidermidis*	*Staphylococcus epidermidis*	**MFP04**
2.S.F1.PD.37.9	*Staphylococcus aureus*	*Staphylococcus aureus*	*Staphylococcus aureus*	**MFP03**
2.S.F2.0D.37.8	***Staphylococcus epidermidis***	***Staphylococcus capitis***	***Staphylococcus epidermidis***	
2.S.F3.0G.28.7	*Pseudomonas fluorescens*	*Pseudomonas fluorescens*	*Pseudomonas fluorescens*	**MFP05**
3.N.F1.FG.37.2	*Paracoccus yeii or Rhodobacteraceae*	*Paracoccus yeei*	*Paracoccus yeii or Rhodobacteraceae*	
3.N.F1.0G.37.6	*Micrococcus luteus*	*Micrococcus luteus*	*Micrococcus luteus*	**MFP02**
3.N.F2.FD.37.12	*Staphylococcus epidermidis*	*Staphylococcus epidermidis*	*Staphylococcus epidermidis*	
3.N.F2.FG.28.12	*Staphylococcus epidermidis*	*Staphylococcus epidermidis*	*Staphylococcus aureus or epidermidis*	
3.N.F2.FG.28.5	*Bacillus cereus*	*Bacillus cereus*	*Bacillus cereus*	MFP01
3.N.F2.FG.28.6	***Actinomyces radingae***	**Microbacterium sp.**	*Microbacterium testaceum*	
3.N.F2.0G.37.2	Roseomonas sp.	*Roseomonas mucosa*	Roseomonas sp.	
3.N.F2.0G.37.3	Brevibacterium sp.	*Brevibacterium casei*	*Brevibacterium linens*	
3.N.F3.FG.28.5	***Gemella haemolysans***	***Staphylococcus capitis***	*Staphylococcus capitis*	
3.N.F3.0G.37.3	Brevibacterium sp.	*Brevibacterium casei*	*Brevibacterium linens*	
3.N.F3.0G.37.7	Brevibacterium sp.	*Brevibacterium casei*	*Brevibacterium spp. casei*	
3.N.F3.PG.37.7	Micrococcus sp.	*Micrococcus luteus*	*Micrococcus luteus*	
3.N.M1.0G.28.1	*Micrococcus luteus*	*Micrococcus luteus*	*Micrococcus luteus*	
3.N.M1.PD.28.1.2	*Staphylococcus equorum*	***Staphylococcus equorum***	***Micrococcus luteus***	

Differences are indicated in bold. Five isolates were included in the LMSM bacterial library under references MFP1 to MFP5.

### Normal skin aerobic cultivable bacterial diversity

A total of 1706 isolates was collected, 573 from 20 to 35 years old normal skins, 638 from 50 to 65 years old normal skins and 495 from 20 to 35 years old sensitive skins (Table 3). A quite identical number of isolates was obtained from male patients and female patients (838 and 868, respectively). The number of isolates found on the different skin areas ranged from 518 on the cheekbones to 238 on the inner elbow. The total percentage of isolates cultivable at 28°C reached 45 ± 3%.

**Table 3:** Comparison of the number of isolates collected in the different groups and skin areas

Phenotypes	Temperature		Gender		Sampling areas				Number of isolates
		
	28°C	37°C	Male	Female	Forehead	Cheekbone	Scapula	Inner elbow	
Normal young skin (20–35 years old)	267	306	304	269	148	159	161	105	573
Normal aged skin (50–65 years old)	334	334	300	338	176	202	127	133	668
Sensitive skin (20–35 years old)	196	299	234	261	162	157	176	ND	495
Total	767	939	838	868	486	518	464	238	1706

**Figure 1:**
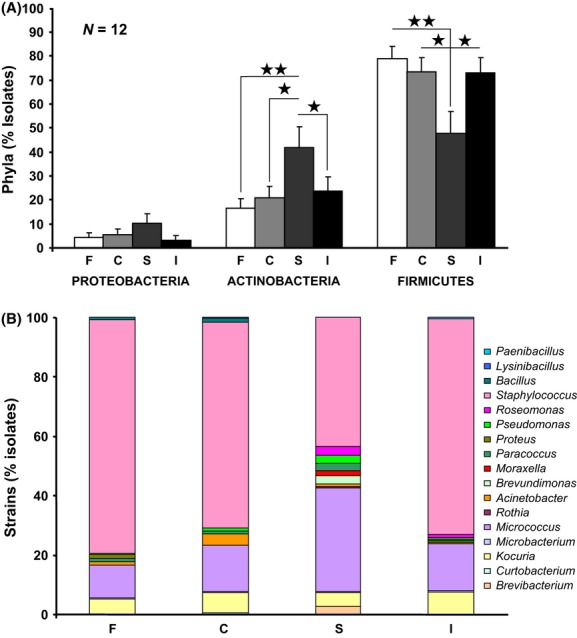
Distribution and relative abundance in the different phyla (A) and genera (B) of bacterial isolates collected on the forehead (F), cheekbones (C), scapula (S), and inner elbow (I) of normal skin patients (⋆*P* < 0.05; *⋆⋆ P* < 0.01)

On this total of 1706 isolates, 134 (7.8%) were leading to a score value under 1.7 by the MALDI-Biotyper technique and were thus impossible to identify. These isolates were excluded from the rest of the study. Hence, this study was finally realized on 1572 isolates that were for a large majority, identified to the species level by mass spectrometry (87.9% isolates with score values >2.0.). All 1572 isolates fell into only three groups, namely Firmicutes, Proteobacteria or Actinobacteria (Fig. 1A). Firmicutes represented 47 ± 9% (on the scapula) to 79 ± 5% (on the forehead) of the total number of isolates and appeared as the predominant phylum on the normal skin. Actinobacteria arrived in second position with a maximum of 42 ± 9% of isolates on the scapula. Proteobacteria were less frequent, their percentages in isolates were ranging from 3 ± 2% on the inner elbow to 10 ± 4% on the scapula. It was possible to point out significant differences in cultivable aerobic bacterial groups between isolates collected on the scapula and on other studied regions: forehead, cheekbones, and inner elbow. Conversely, the differences among these three regions were limited. The MALDI-Biotyper system, allowed the identification of 17 bacterial genera in isolates from the normal skin (Fig. 1B). The population of *Staphylococci* accounted for 78% of identified isolates on the forehead and 68% on the cheekbones. The bacterial diversity was higher on the inner elbow but dominant genera (*Staphylococci*, *Micrococci*, and *Kocuria*) were the same. In samples from the scapula, the population of *Staphylococci* was lower (44%) whereas that of *Micrococci* and Gram-negative bacteria, particularly *Pseudomonas* and *Roseomonas*, was increased.

**Figure 2:**
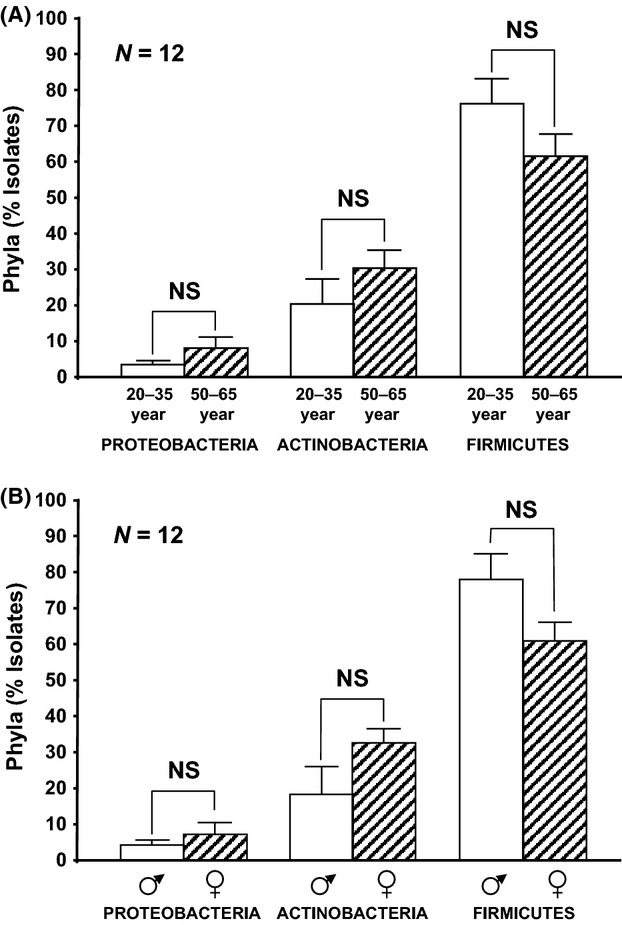
Distribution and relative abundance in the different phyla of bacterial isolates collected on the skin of patients of different ages (A) and sexes (B). NS, nonsignificant.

The relative abundance of the three bacterial groups identified from the skin of young (20–35 years) and older (60–65 years) patients was the same. The percentage of Actinobacteria and Proteobacteria was increased on the skin of 50–65 year-old patients in comparison to the 20–35 years group, but the differences were not significant (Fig. 2A). In parallel, the diversity of bacterial genera identified on older skins was higher than on the younger (16 and 10 genera, respectively). For an unknown reason, the differences between male and female skins were almost the same as between skins of different ages. A higher, but nonsignificant, percentage of Firmicutes was identified in isolates from male skins (Fig. 2B). We also noted an increase in the percentage of Actinobacteria, particularly *Micrococcus,* on female skins. Proteobacteria and bacteria of the genus *Pseudomonas* were also more abundant on female skins.

### Diversity of aerobic cultivable bacteria on normal and sensitive skins

**Figure 3:**
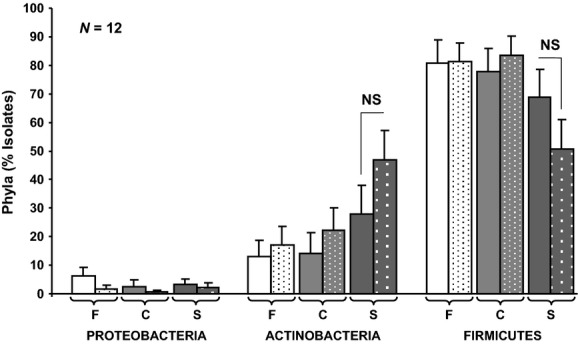
Distribution and relative abundance in the different phyla of bacterial isolates collected on the forehead (F), cheekbones (C), and scapula (S) of normal (clear bars) and sensitive skin (dotted bars) 20–35 year-old.patients. NS, nonsignificant.

As the sensitive skin syndrome was essentially reported in young people (Masako et al. 2005), this study was realized by comparison of isolates from normal and sensitive 20–35 years old skins. A total of 495 isolates was collected on sensitive skin patients. The inner elbow, a region very rarely affected by this syndrome, was not investigated. A decrease in the percentage of Proteobacteria and an increase in Actinobacteria were observed on the forehead, cheekbones, and scapula of sensitive skins, but these differences were not significant (Fig. 3). On the forehead and cheekbones, the percentage of Firmicutes was unchanged. A decrease in Firmicutes was noted in isolates from the scapula of sensitive skin patients, but as previously noted this difference was not significant. The results were not modified when they were analysed as regards the gender of the patients.

**Figure 4:**
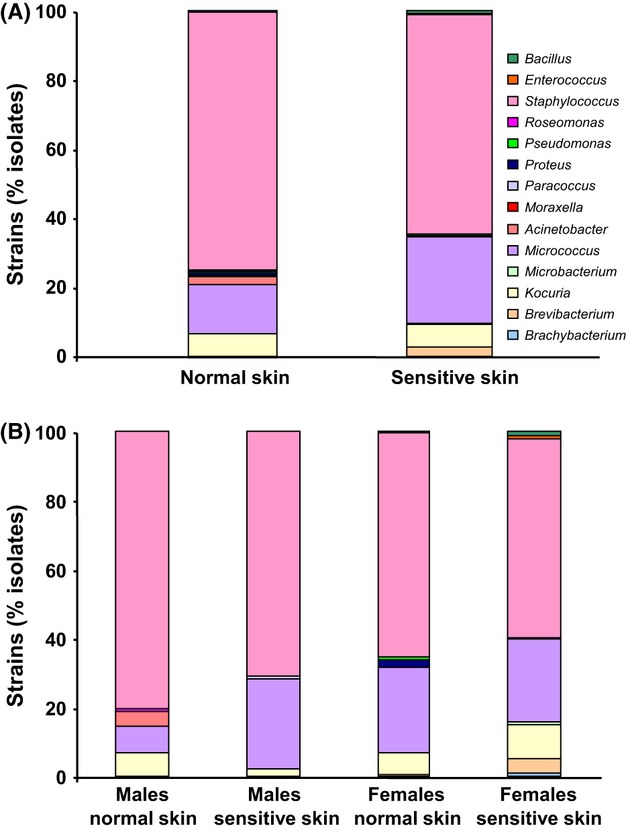
Distribution and relative abundance in the different genera of bacterial isolates collected on the skin of normal and sensitive skin patients (A) and between male and female patients of both skin types (B).

A total of 14 bacterial genera was identified in isolates from normal and sensitive skin patients of both genders. There was no large increase in a bacterial genus or species in any isolate. When the data were separated between normal and sensitive skins, we noted an increase in the percentage of bacteria of the genus *Micrococcus* (+ 48 ± 11%) on sensitive skins (Fig. 4A). In addition, bacteria of the genus Brevibacterium were only detected on the skin of these patients. Conversely, on sensitive skins, bacteria of the genus *Acinetobacter* were totally absent and the percentage of *Kokuria* remained unchanged suggesting that these microorganisms are not involved in the sensitive syndrome. When the data were sorted between genera (Fig. 4B) we remarked that the increase in *Micrococcus* percentage in sensitive skins was due exclusively to isolates collected in male patients (Fig. 4B). In female patients, the *Micrococcus* population was unchanged and it reached the same level as in male sensitive skin patients suggesting that bacteria of the genus *Micrococcus* are not determinant factors in the sensitive skin syndrome. Microorganisms of the genus Brevibacterium and *Enterococcus* were exclusively found in female sensitive skins isolates. Conversely, *Pseudomonas* and *Bacillus*, were only identified on normal skin of female patients. *Acinetobacter* and *Proteus* were exclusively found on normal skins of both sexes. The percentage of *Staphylococci* decreased similarly in male and female sensitive skins (−12%).

## Discussion

A total of 1706 isolates was collected and 1572 were identified to the genus or species level allowing a comparison between the aerobic cultivable bacterial flora of normal and sensitive skins. The collection technique was optimized and the number of isolates in the different groups was homogenous. Of course, the choice of a culture-based technique limits the observations to cultivable and metabolically active bacteria but, as skin is an interface it is actively contaminated by environmental bacteria and genomic approaches also include an undefined number of germs without functional interaction with skin. Other parameters such as skin region, climate, alimentation or even cultural behaviour (Costello et al. 2009; Grice et al. 2009; Rosenthal et al. 2011) also influence the microbial community structure of human skin. Then in the absence of an unbiased method, this study provides a first but partial comparison of the bacteria community on normal and sensitive skins. Another determinant technical parameter of this study was the use of a mass spectrometry-based bacterial identification technique. At the genus level and for clinical species, the performances of the MALDI and 16s rRNA gene sequence-based identification techniques are close (Marko et al. 2012; Nagy et al. 2012) and we observed this was the same for human skin isolates. As we also noted, at the species level the 16s rRNA sequencing technique remains the gold standard (Davies et al. 2012), but as in this study the bacterial communities were differentiated at the groups or genus levels, this limitation was not crucial.

The first interesting result was that 45.4% of skin bacterial isolates started growing at 28°C. This parameter is rarely taken into consideration but it is coherent with the mean surface temperature of the human skin (33°C) (Kopp and Haraldson 1983) that favors the development of microorganisms with a broad growth temperature range. In contrast, whereas in metagenomic studies *Pseudomonas*, and particularly psychrotrophic germs such as *Pseudomonas fluorescens* were detected in abundance (Grice et al. 2008), they were rare in this study. As *P. fluorescens* is ubiquitous, its detection on skin should result from environmental contamination and/or to the detection of DNA sequences from dead bacteria. Alternatively, although *Pseudomonas* have an important fitness potential, auxotrophy to branched chain amino acids, nicotinic acid or other metabolites (Marek-Kozaczuk et al. 2005; Sahu and Ray 2008) that should be provided by the host skin have been shown in fluorescent *Pseudomonas*.

In agreement with previous studies showing that Actinobacteria, Proteobacteria and Firmicutes account for more than 90% of cultivable human skin bacteria (Gao et al. 2007; Costello et al. 2009), in this study all identified isolates fell into one of these groups. However, minor microbial groups such as Bacteroidetes or Cyanobacteria, were totally absent from this study. This could be explained by differences in sampling procedure but more probably by the fact that anaerobic germs were not cultured and that 7.8% of isolates corresponding to nonidentified strains (mass spectrometry score values <1.7) were excluded. This should also explain some differences noted at the genus level. Indeed, Actinobacteria identified in this study were essentially of the genus *Micrococcus,* whereas Kong (2011) reported that on the face, Actinobacteria are essentially microaerophile germs such as Propionibacteria and Corynebacteria. As a consequence, Firmicutes (*Staphylococci*) appeared overrepresented in comparison to other studies (Gao et al. 2007; Costello et al. 2009).

The approach employed in this study allowed to reveal significant regional distribution of bacterial groups on the different skin areas. These differences, particularly between the scapula and other regions, should be explained by variations of dryness and mean temperature. Conversely, although marginal variations were observed between patients of different ages and sexes there was no significant differences among these groups and that was allowing a comparison of the skin bacterial community of mixed panels. As the sensitive skin syndrome was essentially reported in young people (Masako et al. 2005), we only compared isolates from 20 to 35-year old patients. Samples collected on the inner elbow were also excluded from the comparison since, as observed in our group, this area is rarely affected by the syndrome. Proteobacteria, remained in very limited number on sensitive skins. Conversely, a marginal and nonsignificant increase in Actinobacteria was noted on the two face areas and on the scapula but these differences appeared directly correlated with an increase in bacteria of the genus *Micrococcus* in male patients. The population of bacteria of the species *Kocuria* evolved in opposite sense between male and female sensitive skins. Other genera, such as Brevibacterium, *Enterococcus*, *Pseudomonas* or *Bacillus* were only found on the skin of male patients or female patients. Except if we hypothesize that the sensitive skin syndrome should be provoked by different bacterial species in men and women we can exclude these germs as determinants of this disorder. Acinetobacter and *Proteus* were not detected in samples from sensitive skin patients and the percentage of *Staphylococci* even decreased. Consequently, although a total of 495 bacterial isolates was collected on sensitive skin patients, there was no detectable correlation between a phylum, a genus or even a dominant bacterial species and the sensitive skin phenotype. This is in contradiction with data showing that a partial elimination of the skin bacterial population is improving itch and pain in sensitive skin patients (Masako et al. 2005; Berardesca et al. 2009). In order to understand these results, it should be essential to remember that bacterial virulence is highly variable (Henderson and Martin 2011) and that a same bacterium can switch from harmless to virulent under the effect of environmental factors, including host communication molecules (Lesouhaitier et al. 2009). As skin is the body largest neuroimmunoendocrine organ (Roosterman et al. 2006), cutaneous bacteria are exposed to a multitude of factors potentially able to stimulate their virulence. Another hypothesis should be the release of irritant metabolites by sensitive skin specific bacterial strains that remained undetectable in this study. Indeed, important differences in toxicity are frequently observed between bacterial strains of a same species (Chapalain et al. 2008). These hypotheses remain to be investigated.

Taken together, we did not observe the emergence of a dominant bacterial group, genus or species on the skin of sensitive skin patients. These results apparently contradict the data suggesting a role of the bacterial community in the sensitive skin syndrome. However, as this study only considered aerobic cultivable bacteria, we cannot exclude an involvement of microaerophile or noncultivable germs. Alternatively, specific bacterial strains or factors produced by skin could trigger a global increase in bacterial virulence.
